# Supplementary material for: Identification of mammalian transcription factors that bind to inaccessible chromatin
Source: Nucleic Acids Res. 2023 Jul 24;51(16):8480–95. doi: 10.1093/nar/gkad614 (PMC10484684; doi:10.1093/nar/gkad614)
Supplement: gkad614_Supplemental_Files [file gkad614_supplemental_files.zip › Table_S4_R1.docx]

**Table S4.** Summary of predicted/validated pioneer factors in humans in the literature.

| TF name | Reference |
| --- | --- |
| FoxA1/2 | reviewed in (1) |
| Oct3/4, Pou5f3 | reviewed in (1) |
| Sox2 | reviewed in (1) |
| Klf4 | reviewed in (1) |
| Ascl1 | reviewed in (1) |
| Pax7 | reviewed in (1) |
| PU.1 | reviewed in (1) |
| GATA4 | reviewed in (1) |
| GATA1 | reviewed in (1) |
| CLOCK:BMAL1 | reviewed in (1) |
| P53 | reviewed in (1) |
| Pbx1 | reviewed in (2) |
| Gro/TLE/Grg | (3) |
| AP-1 (Jun/Fos) | (4) |
| CREB1 | (5) |

1. Iwafuchi-Doi,M. and Zaret,K.S. (2014) Pioneer transcription factors in cell reprogramming. *Genes Dev.*, **28**, 2679–2692.

2. Grebbin,B.M. and Schulte,D. (2017) PBX1 as pioneer factor: A case still open. *Front. Cell Dev. Biol.*, **5**, 9.

3. Sekiya,T. and Zaret,K.S. (2007) Repression by Groucho/TLE/Grg Proteins: Genomic Site Recruitment Generates Compacted Chromatin In Vitro and Impairs Activator Binding In Vivo. *Mol. Cell*, **28**, 291–303.

4. Biddie,S.C., John,S., Sabo,P.J., Thurman,R.E., Johnson,T.A., Schiltz,R.L., Miranda,T.B., Sung,M.H., Trump,S., Lightman,S.L., *et al.* (2011) Transcription Factor AP1 Potentiates Chromatin Accessibility and Glucocorticoid Receptor Binding. *Mol. Cell*, **43**, 145–155.

5. Sherwood,R.I., Hashimoto,T., O’Donnell,C.W., Lewis,S., Barkal,A.A., Van Hoff,J.P., Karun,V., Jaakkola,T. and Gifford,D.K. (2014) Discovery of directional and nondirectional pioneer transcription factors by modeling DNase profile magnitude and shape. *Nat. Biotechnol. 2013 322*, **32**, 171–178.
